# Diversion Colitis: Macro and Microscopic Findings after Probiotics Stimulation

**DOI:** 10.3390/biology10040303

**Published:** 2021-04-06

**Authors:** Ángela Rodríguez-Padilla, Germán Morales-Martín, Rocío Pérez-Quintero, Juan Gómez-Salgado, Ricardo Rada-Morgades, Carlos Ruiz-Frutos

**Affiliations:** 1 Department of General Surgery, Infanta Elena University Clinical Hospital, 21080 Huelva, Spain; angelar.rodriguez.sspa@juntadeandalucia.es (Á.R.-P.); german.morales.sspa@juntadeandalucia.es (G.M.-M.); 2 Department of General Surgery, Juan Ramón Jiménez University Clinical Hospital, 21005 Huelva, Spain; rocio.perez.quintero.sspa@juntadeandalucia.es (R.P.-Q.); ricardo.rada.sspa@juntadeandalucia.es (R.R.-M.); 3 Department of Sociology, Social Work and Public Health, Faculty of Labour Sciences, University of Huelva, 21007 Huelva, Spain; frutos@uhu.es; 4 Safety and Health Postgraduate Programme, Universidad Espíritu Santo, Guayaquil 092301, Ecuador

**Keywords:** diversion colitis, colonoscopy, efferent loop stimulation, ileostomy closure, probiotics, microbiome therapeutics

## Abstract

**Simple Summary:**

The observations presented in this study conclude that the preoperative stimulation with probiotics of the efferent loop through the dysfunctional bowel, to allow the slow infusion, can have a reducing effect on the endoscopic and histopathological alterations of diversion colitis. This procedure may be an alternative treatment to resolve the inflammation in patients where the surgical option is not feasible or available.

**Abstract:**

The use of a loop ileostomy as the defunctioning procedure of choice to protect a distal colonic anastomosis causes histological and endoscopic changes in the intestinal mucosal architecture, which have been related to chronic inflammation and changes in the microflora that consequently impact the intestinal structure and function following fecal stream diversion. The aim of this study was to evaluate the histological and endoscopic changes on the colonic mucosa in patients with diversion colitis after stimulation of the efferent loop with probiotics prior to closure of the protective ileostomy. A prospective, randomized, double-blind, controlled study was designed. All patients who underwent surgery for colorectal carcinoma with protective ileostomy between January 2017 and December 2018 were included. These patients were pending reconstructive surgery and were diagnosed with endoscopic and histological diversion colitis. Divided into two groups, a group stimulated with probiotics (SG) and a control group (CG). 34 cases and 35 controls were included in the study. Histological and endoscopic changes were evaluated after stimulation, after restorative surgery and during the short-term follow-up after surgery. A decrease in endoscopic pathological findings (mucosal friability, mucous erosions, polyps, edema, erythema and stenosis) and in histological findings (follicular hyperplasia, eosinophils, cryptic abscesses, lymphocyte infiltration, plasma cell infiltration and architecture distortion) was observed in SG. These results were statistically significant with a *p* < 0.001. The stimulation of the efferent loop of the ileostomy in patients with diversion colitis produced a decrease of the endoscopic and histological severity of colitis in the short term.

## 1. Introduction

Probiotics were defined as live microorganisms that improve the host’s health when administered in adequate amounts [1,2]. These microorganisms and their metabolic products have been suggested as food supplements to achieve a healthier intestinal homeostasis and, also, as a treatment for pathologies with an important inflammatory component. In recent years, multiple studies have been published which explain the use of probiotics and their effect on the microbiota flora when applied to different pathologies such as diabetes mellitus, obesity, inflammatory bowel disease, colitis and even psychological disorders [3,4,5,6,7,8,9,10]. Depending on the strains included in probiotics, with different biochemical and immunological properties, the modulating effect of inflammation was evaluated in vitro and in vivo [11]. These biological effects are closely related to the microbiota metabolism and the ability to colonize the gastrointestinal tract [7]. Probiotics interact with the intestinal mucosa, reducing the molecular production of proinflammatory substances [1]. Live bacteria, such as Lactobacillus, Bifidobacterium, and Enterococcus, present antimicrobial and immunomodulatory activity and improve the intestinal barrier [3]. This immunomodulatory effect is what is needed in order to reduce the level of diversion colitis [12,13,14]. Currently available probiotics, aimed at other pathologies with inflammatory conditions, produce a modulating effect in a transitory and limited way. This time-limited effect is also essential to reduce the level of diversion colitis, since after reconstruction the effect of probiotics tends to disappear [15,16].

In patients suffering from colorectal cancer who have undergone partial colonic resection, the creation of a temporary ileostomy causes a defunctionalization of the colon [15]. Consequently, the microflora within the defunctioned colon is less diverse and convergence between defunctioned microbiota profiles was observed; additionally, a chronic inflammation appears, with endoscopic and histological changes compatible with diversion colitis (DC) [17,18].

Diversion colitis was described by Glotzer et al. in 1981 [19]. It is characterized by an inflammation of the large bowel mucosa that mimics idiopathic inflammatory bowel disease [15]. It has an incidence between 50–91% [15,16]. It can manifest symptoms such as abdominal or pelvic pain, mucous discharge, tenesmus, fever, and rectal bleeding in the most severe cases, although up to 30% of patients remain asymptomatic [16]. 52% of patients with DC show mild inflammation, 44% moderate and 4% severe [3,20], which represents an annual incidence close to 120,000 patients/year. DC is still a problem and probably will remain so in the future, which impairs the quality of life of a significant number of patients [15,16]. DC shows a wide spectrum of endoscopic and histopathological changes, most importantly in the distal segments of the colon.

The endoscopic findings described are: friable mucosa, edema, erythema, appearance of polyps, ulcers, stenosis and histological findings such as lymphoid follicular hyperplasia, infiltration of the lamina propria by lymphocytes, eosinophils, the appearance of plasma cells, architectural disruption, and the appearance of crypt abscesses [15,21].

The definitive treatment is the restoration of the continuity of the digestive tract [15,16]. Pharmacological treatments using instillation techniques with short-chain fatty acids, mesalazine, fiber or corticosteroids are reserved for patients who are not candidates for surgical treatment or for stimulation of the efferent loop prior to surgery [22,23,24]. Stimulation with probiotics prior to closing the protective stoma would allow the dysfunctional colon segment to be repopulated, which would reduce diversion colitis [3,15,16]. The objective of this study was to evaluate the histological and endoscopic changes in the colonic mucosa in patients with diversion colitis after stimulation of the efferent loop with probiotics and prior to closure of the protective ileostomy.

## 2. Materials and Methods

### 2.1. Study Design

Prospective, randomized, multicenter, double-blind study, comparing two groups of patients operated for colorectal carcinoma with protective ileostomy. One group includes patients treated with probiotic stimulation of the efferent loop prior to transit reconstruction surgery; the other control group was stimulated without any substance.

### 2.2. Sample Size

The sample size was calculated according to the diversion colitis endoscopic and histological findings published in previous studies [16,24]. Assumed reduction in macroscopic and microscopic pathological findings was 50% (100–50%). With a loss adjustment of 15%, it was necessary to have 30 patients per group. We recruited 34 patients for the stimulated group and 35 patients for the control group for a statistical level of 95% and a power of 0.8.

### 2.3. Selection of Patients

**Figure 1:**
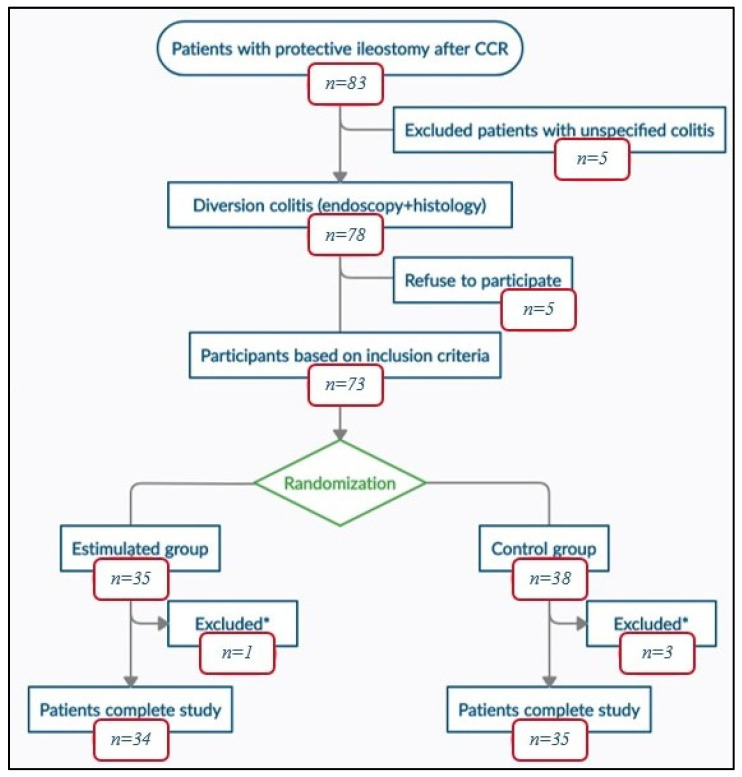
Study flowchart; CRC: Colorectal Cancer; *: excluded patients with anastomotic leak.

Between January 2017 and December 2018, all the patients, from the three participating centers, that were included in the surgical waiting list for temporary stoma closure (coded according to CIE-10) after colorectal carcinoma, were consecutively evaluated to determine their inclusion in the study. The flowchart for the selection of the study patients is depicted in Figure 1.

The inclusion criteria were being over 18 years of age, having protective ileostomy after colorectal carcinoma surgery free of disease, with endoscopic and histological confirmation of diversion colitis and having signed the informed consent. As exclusion criteria: being under 18 years of age, clinical history, and histological confirmation of inflammatory bowel disease with colorectal involvement, and refusal to participate in the study. Abandonment criteria: loss during follow-up, exitus, and anastomotic dehiscence after reconstruction surgery.

### 2.4. Endoscopic and Histopathological Examination

Colonoscopies with random biopsies were performed on all patients to exclude those who did not have colitis and to establish the severity index. Three endoscopists performed the endoscopies with a Silver Scope Storz^®®^ colonoscope (Karl Storz SE & Co. KG, Tuttlingen, Germany). The endoscopic findings were expressed according to the scoring system of Harig et al. [20] including erythema, edema, friability, polypus, granularity, stenosis and erosions. Every finding was scored in a scale from 0 to 1 or from 0 to 3, and the grade of colitis was obtained by adding these scores. The total score was validated as absent, mild, moderate, or severe. The histopathological analysis was assessed by three independent histopathologists. Samples of colon were fixed in buffered formalin and stained with hematoxylin and eosin (H&E). In the same way as in the endoscopy, DC was diagnosed by evaluating the appearance of follicular lymphoid hyperplasia, eosinophilic, lymphocytes and plasma cells infiltrations and crypt architecture distortion. Every finding was scored in a scale from 0 to 1 or from 0 to 3, obtaining the grade of colitis by adding the results. The total score was validated as absent, mild, moderate, or severe.

Both endoscopic and histopathological studies were performed after stimulation and 3 months after intestinal continuity reconstruction.

### 2.5. Randomization and Intervention

After confirming the diagnosis and excluding patients who did not meet the selection criteria, randomization was performed creating two groups by using a computer-generated sequence on EPIDAT (Statistical software EPIDAT version 4.2. Consellería de Sanidade, Xunta de Galicia, España; Organización Panamericana de la Salud (OPS-OMS); Universidad CES, Colombia):

Stimulation group (SG): preoperative stimulation with probiotics of the distal limb of the ileostomy loop was performed during the 20 days prior to surgery every second day. During the process and after it, the patient himself registered the appearance of symptoms after each stimulation session: abdominal pain, emission of gas and stool. A sterile Foley catheter No.14 Ch connected to an infusion set was introduced through the defunctioned bowel. This was done to allow slow infusion of a solution of 4.5 mg of probiotics diluted in 250 mL of 0.9% physiological saline for 20–30 min. Each preparation was made under sterile conditions and maintaining the cold chain. Vivomixx^®®^ lyophilized live bacteria, marketed by MENDES, S.A, contained 4.5 × 10^11^ of live bacteria in each preparation:

◦ Four strains of *Lactobacillus*:
  ■ Lactobacillus acidophilus DSM 24735^®®^
  ■ Lactobacillus plantarum DSM 24730^®®^
  ■ Lactobacillus paracasei DSM 24733^®®^
  ■ *Lactobacillus delbrueckii* subsp. bulgaricus DSM 24734^®®^
◦ Three strains of *Bifidobacterium*:
  ■ Bifidobacterium breve DSM 24732^®®^
  ■ Bifidobacterium longum DSM 24736^®®^
  ■ Bifidobacterium infantis DSM 24737^®®^
◦ One strain of *Streptococcus*
  ■ Streptococcus thermophilus DSM 24731^®®^

Control group (CG): exactly the same procedure was carried out, but with the closed infusion set. During the process and after it, the patient himself registered the appearance of symptoms after each stimulation session: abdominal pain, emission of gas and stool.

After ten stimulation sessions, 24 h before surgery, a colonoscopy with biopsy was performed on all patients, re-quantifying the index of severity of endoscopic and histological diversion colitis.

### 2.6. Surgery and Follow-Up

The reconstruction surgery was carried out by three expert surgeons from the Colorectal Surgery Department. A parastomal incision was made and carried out sharply into the peritoneal cavity. The anastomosis was lateral-lateral, either manual or mechanical, according to the decision of the surgeon. Every surgeon could decide whether to change to a median laparotomy procedure or not. Complications or events happened during surgery were recorded in the surgical procedure protocol. General anesthesia was given to all patients and, after extubation and stabilization in the postoperative recovery room, they went directly to the hospitalization ward.

Follow-up during hospitalization was carried out by the team of the Colorectal Surgery department of every center, recording any postoperative complications. Patients were discharged from the hospital after re-establishing intestinal transit, adequate oral tolerance and stool control, recording the length of their hospital stay.

Follow-up after hospitalization was carried out by the Colorectal Surgery team in the first, third and sixth postoperative months. These evaluations were performed by the colorectal surgeon who had performed the surgical intervention in each patient. Any symptomatology related to the intervention was recorded, with special monitoring of abdominal pain, number and control of stools, mucous discharge, tenesmus and rectorrhagia. After completing a three-month follow-up, a colonoscopy with biopsies was performed to evaluate the presence and grade of DC.

### 2.7. Masking

To ensure masking of the patients, all underwent the same diagnostic procedure. During the stimulation sessions, both the solution with probiotics and the infusion set were covered by an opaque protective envelope, which prevented from observing the color and transparency of the fluid, or whether the system was open or closed. The stimulation sessions were performed by a single surgeon, who was also in charge of preparing the dilution.

The endoscopist, the pathologist and the surgeon who performed the surgical intervention and the follow-up, as well as the surgeons who participated in the hospitalization process after the surgery, did not know whether the patient had received probiotics or not.

### 2.8. Assessment Criteria

The main endpoint was the decrease or disappearance of endoscopic and histological findings in the short-term follow-up, caused by stimulation of the efferent loop with probiotics. The secondary endpoints were the correlation of endoscopic and histological severity with DC symptoms and its decrease or disappearance after stimulation with probiotics and in the short-term follow-up after reconstructive surgery (3 months).

### 2.9. Statistical Analysis

A descriptive univariate analysis of sociodemographic and clinical variables was performed. The Kolmogorov-Smirnov test was used to verify the normality of the quantitative variables. To describe the quantitative variables, the mean and standard deviation were used, and the median and interquartile range for those variables that did not follow a normal distribution. For qualitative variables frequencies and percentages were used. Afterwards, to verify the main objectives, a bivariate analysis was performed. A contrast test of proportions based on the Chi-square test was used in order to determine whether stimulation of the efferent loop with probiotics prior to the closure of the protective ileostomy reduced the level of diversion colitis, macroscopic and microscopic findings, as well as its relationship with the appearance of symptoms of diversion colitis. To quantify its level of association, Cramer’s Phi and V coefficients were calculated. In order to correlate quantitative variables, the Spearman’s rank correlation coefficient was used. A *p* < 0.05 was considered to be significant. Statistical analyses were performed using the statistical program SPSS^®®^ version 24.0 (IBM, Armonk, NY, USA), with the support of calculation tools provided by the software Microsoft Excel and R.

### 2.10. Ethical Aspects

The project was performed according to the guidelines of the Declaration of Helsinki, with the authorization of the Ethics Coordinating Committee for Biomedical Research of Andalusia, Spain, registered with the project number 2017/331191354. Written informed consent was requested to participate in the study, giving details of both the study objectives and the methodology to be followed. The data was kept anonymous, maintaining the confidentiality and anonymity of the participants.

## 3. Results

### 3.1. Study Population

Between January 2017 and December 2018, 83 disease free patients with protective ileostomy after colorectal carcinoma resection were reviewed and included in the surgical waiting list for intestinal transit reconstruction. 78 of them met the endoscopic and histological criteria for diversion colitis diagnosis and 73 patients were finally randomized into two groups, intervention (*n* = 35) and control (*n* = 38). 69 patients completed the study, 1 of them from SG and 3 from CG abandoning the study due to anastomotic leak.

There were no significant differences between SG and CG in terms of sociodemographic, clinical or surgical variables (Table 1). Symptoms of DC were observed in 70.6% of patients in SG (abdominal pain in 44.1%, mucous rectal discharge in 61.7%, rectal tenesmus in 14.7% and bleeding in 5.9%) and in 60% of patients in CG (abdominal pain in 51.4%, mucous rectal discharge in 40%, rectal tenesmus in 5.7% and bleeding in 11.4%).

### 3.2. Main Endpoints

#### 3.2.1. Macroscopic Findings

**Figure 2:**
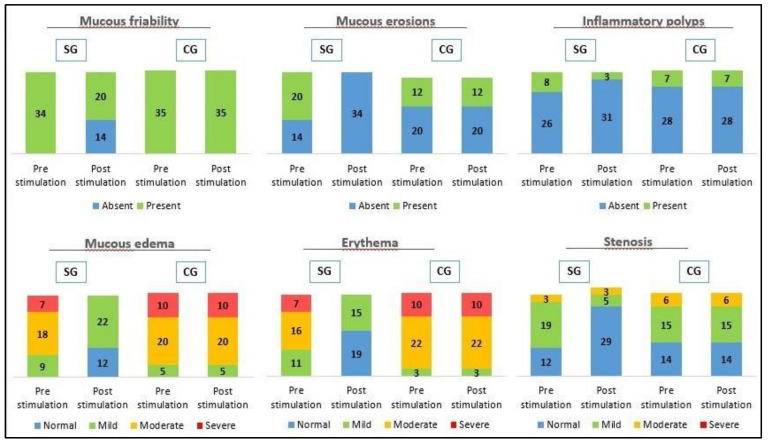
Macroscopic findings by endoscopy before and after stimulation in CG and SG in diversion colitis. A statistically significant decrease in endoscopic pathological findings (mucosal friability, mucous erosions, polyps, edema, erythema and stenosis) were observed in SG, with a *p* < 0.001.

The effects of probiotic stimulation are shown in Figure 2. A homogeneous distribution between SG and CG was observed in the pre-stimulation phase. Friability of the colonic mucosa was seen in 100% of patients in both CG (*n* = 35) and SG (*n* = 34). Differences were found in the post-stimulation phase where friability of the colonic mucosa was seen in 100% of patients in CG compared to 58.8% in SG (*n* = 20), where this symptom disappeared in 41.2% of patients (*n* = 14) with *p* < 0.001 and a Phi and V Cramer coefficient of 0.948. Friability of the colonic mucosa had already disappeared in both groups after reconstructive surgery in the short-term follow-up.

The presence of ulcers in the colonic mucosa was found in 34.3% of patients in CG (*n* = 12) and 58.8% in SG (*n* = 20). These lesions were observed in colonoscopy after stimulation with probiotics in 34.4% of patients in CG, unlike SG, in which it disappears with *p* < 0.001 and a Phi and V Cramer coefficient of 0.693. The ulcers disappeared in both groups after reconstructive surgery in the short-term follow-up.

Inflammatory polyps were present in 20% of patients in CG (*n* = 7) and 23.5% in SG (*n* = 8). After stimulation with probiotics polyps were still present in 20% of CG whether they reduced to 8.8% of SG (*n* = 3) with *p* < 0.001 and a Phi and V coefficient of Cramer 0.781. The inflammatory polyps disappeared in both groups after reconstructive surgery in the short-term follow-up.

Mucosal edema was present in 100% in both CG (*n* = 35) and SG (*n* = 34). The distribution of CG according to endoscopic severity was 5 mild (14.3%), 20 moderate (57.1%) and 10 severe (28.6%); contrasting with SG, 9 mild (36.5%), 18 moderate (52.9%), and 7 severe (20.6%). Mucosal edema was maintained in colonoscopy after stimulation with probiotics in 100% of CG with the same distribution of severity, compared to SG, 22 mild (64.7%) and no edema in 12 patients (35.3%), with a *p* < 0.001 and a Phi and V Cramer coefficient of 0.670. The edema had disappeared in 85.7% in CG (*n* = 30) and 91.2% in SG (*n* = 31). In the short-term follow-up, after reconstructive surgery, we observed mild edema in 14.3% in CG (*n* = 5) and 8.8% SG (*n* = 3), with a *p* = 0.479.

Mucosal erythema was present in 100% of both, CG (*n* = 35) and SG (*n* = 34). The distribution of CG according to endoscopic severity was 3 mild (8.6%), 22 moderate (62.9%) and 10 severe (28.6%); in contrast to SG, 11 mild (32.3%), 16 moderate (47.1%), and 7 severe (20.6%). Mucosal erythema was maintained in colonoscopy after stimulation with probiotics in 100% of CG with the same distribution of severity, compared to SG, 15 patients (44.1%) and no erythema in 19 patients (55.9%), with a *p* < 0.001 and a Phi and V Cramer coefficient of 0.976. In the short-term follow-up, after reconstructive surgery, we observed erythema had disappeared in both groups.

Bowel stenosis was present in 60% in CG (*n* = 21) and 64.7% SG (*n* = 22). The distribution of CG according to endoscopic severity was 15 mild (42.9%) and 6 moderate (17.1%), compared to SG, 19 mild (55.9%) and 3 moderate (8.8%). Bowel stenosis was maintained in colonoscopy after stimulation with probiotics in 60% in CG with the same distribution of severity, compared to SG, 5 patients (14.7%) and no stenosis in 29 patients (85.3%), with a *p* < 0.001 and a Phi and V Cramer coefficient of 0.842. In the short-term follow-up, after reconstructive surgery, we observed stenosis had disappeared in 97.1% in CG (*n* = 34) and in 100% in SG (*n* = 34), with a *p* = 0.321.

#### 3.2.2. Microscopic Findings

**Figure 3:**
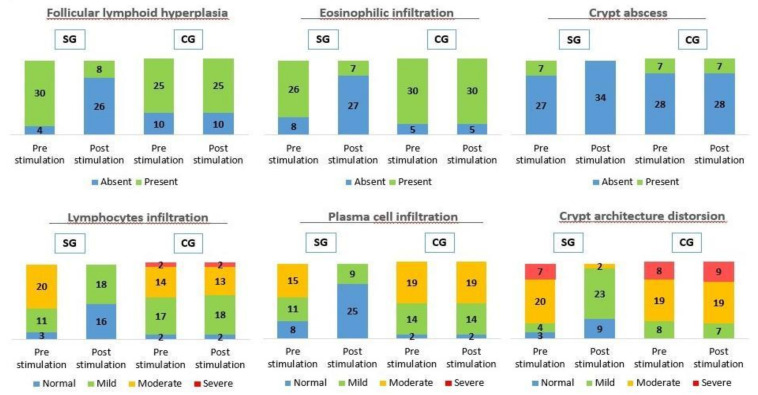
Microscopic findings by histological study before and after stimulation in CG and SG in diversion colitis. A statistically significant decrease in histologic pathological findings (follicular hyperplasia, eosinophils, cryptic abscesses, lymphocyte infiltration, plasma cell infiltration and architecture distortion) were observed in SG, with a *p* < 0.001.

The effects of probiotic stimulation were depicted in Figure 3. A homogeneous distribution between SG and CG was observed in the pre-stimulation phase. Lymphoid follicular hyperplasia was present in 71.4% of patients in CG (*n* = 25) and 88.2% in SG (*n* = 30) and was maintained after stimulation with probiotics in 71.4% in CG compared to 23.5% in SG (*n* = 8), being absent in 76.5% in SG (*n* = 26), with a *p* < 0.001 and Phi and V Cramer coefficient of 0.683. In the short-term follow-up lymphoid follicular hyperplasia had already disappeared after reconstructive surgery in both groups. Eosinophils were present in 85.7% in CG (*n* = 30) and 76.5% in SG (*n* = 26) and it was maintained after stimulation with probiotics in 85.7% in CG compared to 20.6% in SG (*n* = 7), being absent in 79.4% in SG (*n* = 27), with a *p* < 0.001 and Phi and V Cramer coefficient of 0.518. In the short-term follow-up eosinophils were only present in 2.9% after reconstructive surgery in both groups, with a *p* = 0.182. Crypt abscesses were present in 20% in CG (*n* = 7) and 20.6% in SG patients (*n* = 7) and it was maintained after stimulation with probiotics in 20% in CG, less when compared to SG in which were absent in 100% of patients (*n* = 34), with a *p* < 0.001 and Phi and V Cramer coefficient of 0.666. In the short-term follow-up crypt abscesses had already disappeared after reconstructive surgery in both groups. Lymphocytic infiltration of the lamina propria was present in 94.3% of patients in CG (*n* = 33) and 91.2% in SG (*n* = 31). The distribution of CG according to endoscopic severity was 17 mild (48.6%), 14 moderate (40%) and 2 severe (5.7%) while in SG it was 11 mild (32.4%) and 20 moderate (58.8%). Lymphocytic infiltration of the lamina propria was maintained in 94.3% in CG with the same distribution after stimulation with probiotics, compared to SG with 18 mild (52.9%) and absence in 16 patients (47.1%), with a *p* < 0.001 and Phi and V coefficient of Cramer 0.692. In the short-term follow-up lymphocytic infiltration of the lamina propria had disappeared in 85.7% in CG (*n* = 30) and 100% in SG (*n* = 34) after reconstructive surgery, persisting in a mild form in 14.3% in CG (*n* = 5), with a *p* = 0.004 and Phi and V Cramer coefficient of 0.442. Plasma cell infiltration was present in 94.3% in CG (*n* = 33) and 76.5% in SG (*n* = 26). The distribution of CG according to endoscopic severity was 14 mild (40%) and 19 moderate (54.3%). However, it was observed in SG 11 mild (32.4%) and 15 moderate (44.1%). Plasma cell infiltration was maintained with the same distribution after stimulation with probiotics in 94.3% in CG compared to SG with 9 mild (26.5%) being absent in 25 in SG (73.5%), with a *p* < 0.001 and Phi and V Cramer coefficient 0.771. In the short-term follow-up plasma cell infiltration had disappeared in 94.3% in CG (*n* = 33) and 97.1% in SG (*n* = 33) after reconstructive surgery, persisting mild infiltration by lymphocytes in 5.7% in CG (*n* = 2) and 2.9% in SG (*n* = 1), with a *p* = 0.190. Destructuring of the crypt architecture was present in 100% in CG (*n* = 35) and 91.2% in SG (*n* = 31). The distribution of CG according to endoscopic severity was 8 mild (22.9%), 19 moderate (54.3%) and 8 severe (22.9%), however in SG 4 mild (11.8%), 20 moderate (58.8%) and 7 severe (20.6%) was observed. Destructuring of the crypt architecture was maintained, after stimulation with probiotics, in 100% in CG with the same distribution, compared to SG with 23 mild (67.6%), 2 moderate (5.9%) and absence in 9 patients (26.5%), with a *p* < 0.001 and Phi and V Cramer coefficient of 0.911. In the short-term follow-up destructuring of the crypt architecture had disappeared in 85.7% in CG (*n* = 30) and 100% in SG (*n* = 34), persisting mild alteration of the architecture in 14.3% in CG (*n* = 5) after reconstructive surgery, with a *p* = 0.002 and Phi and V Cramer coefficient of 0.471.

### 3.3. Secondary Endpoints

We found a homogeneous distribution in the diversion colitis degree in pre-stimulation phase between SG and CG (*p* = 0.911), with 9 patients with severe diversion colitis in both groups (26.5% SG vs 25.7% CG), 23 patients with moderate diversion colitis in both groups (67.6% SG vs 65.7% CG) and 2 patients with mild diversion colitis in SG (5.9%) versus 3 patients in CG (8.6%). The associated symptoms were abdominal pain, mucous discharge, urgency, and rectal bleeding (Table 1). Abdominal pain was present in 44.1% of patients in SG (*n* = 15) and 51.4% in CG (*n* = 18). Mucous discharge was present in 61.7% of patients in SG (*n* = 21) and 40% in CG (*n* = 14). Tenesmus appeared in 14.7% in SG (*n* = 5) and 5.7% in CG (*n* = 2). Rectorrhagia was observed in 5.9% in SG (*n* = 2) and 11.4% in CG (*n* = 4).

In the post-stimulation phase, CG maintains its distribution with 9 patients with severe diversion colitis (25.7%), 23 patients with moderate diversion colitis (65.7% CG) and 3 patients with mild diversion colitis (8.6%); on the other hand, SG shows no patients with severe diversion colitis (*n* = 0), 3 patients with moderate diversion colitis (8.8% CG), 19 patients with mild diversion colitis (55.9%) and 12 patients with normal endoscopic and histological findings (35.3%), achieving a *p* < 0.001 and a Phi and V Cramer coefficient of 0.834. After stimulation and reconstructive surgery, DC symptoms were present in 100% in CG (*n* = 35) compared to 14.7%in SG (n = 5) with a *p* < 0.001 and Phi and V coefficient of Cramer 0.789. The associated symptoms in CG were diarrhea in 85.7% (*n* = 30), abdominal pain in 57.1% (*n* = 20), urgency in 34.3% (*n* = 12) and rectal bleeding in 5.7% (*n* = 2). The associated symptoms in SG were abdominal pain in 11.7% (*n* = 4) and tenesmus in 5.9% (*n* = 2). In the post-stimulation phase, CG maintains its distribution with 9 severe DC (25.7%), 23 moderate DC (65.7% CG) and 3 mild DC (8.6%); on the other hand, SG shows no patients with severe DC (*n* = 0), 3 patients with moderate DC (8.8% CG), 19 patients with mild DC (55.9%) and 12 patients with normal endoscopic findings (35.3%), achieving a *p* < 0.001 and Phi and V Cramer coefficient of 0.883.

Finally, during the short-term follow-up after reconstructive surgery, symptoms were present in CG with 28.6% (*n* = 10) compared to 71.4% who were asymptomatic (*n* = 25). SG showed 100% asymptomatic patients after surgery, with *p* < 0.001 and a Phi and V Cramer coefficient of 0.406. The persistent symptoms in CG were diarrhea 28.6% (*n* = 10), abdominal pain 22.8% (*n* = 8) and tenesmus 5.7% (*n* = 2).

## 4. Discussion

In our study, macroscopic and microscopic alterations associated with DC dysbiosis were observed in both CG and SG without observing a statistically significant relationship between severity, symptoms, or time since the creation of the stoma, findings similar to those published in previous studies [15,16,25,26,27,28]. There does seem to be a significantly strong relationship between stimulation with probiotics and the decrease in endoscopic and histological severity on one hand, and between, stimulation with probiotics and the decrease in symptoms after reconstructive surgery on the other. In our series, symptoms associated with DC were presented in 100% of patients in CG compared to 14.7% in SG with a Phi and V Cramer coefficient that indicates a relatively strong relationship between stimulation with probiotics and the appearance of symptoms after the closure of the ileostomy; a fact that we believe is related to the decrease in dysbiosis in the defunctionalized segment of the colon and which was maintained in the short term. This dysbiosis activates metabolic products that produce an immune response, causing an attack on the colonic mucosa and affecting the function of the regulatory immune cells that normally promote its homeostasis [25]. Its decrease causes an improvement in structural changes, such as atrophy of villi in the defunctionalized limb, and in consequence produces a loss of smooth muscle area and a reduced isometric contractility with loss of the intestinal absorption capacity [18]. Intestinal dysbiosis has been linked to the pathogenesis of numerous chronic diseases, such as inflammatory bowel disease, and has been studied in order to identify its correction obtaining variable results [4,26].

Most of the clinical research on DC was carried out during the 90’s and early 2000’s with the aim of identifying the molecular mechanisms that triggered this disease, its endoscopic and histological characteristics, and its relationship with the microbiota alteration [21,29,30,31,32]. In the last two years, two studies have been published regarding intestinal microbiota, Mishima [4] and Knox [33]; to which we must add, on the one hand, the latest systematic review on diversion colitis published by Kabir et al. [16] in 2014 that reviews a total of 3305 articles, eventually including 35 studies, and analyses the pathophysiology, clinical presentation and treatment of diversion colitis which concludes that there is a great variability of forms of presentation and a single definitive treatment, which is the reconstruction of the transit. Results on preoperative stimulation prior to the closure of the protective ileostomy published by Rombey et al. [23] in 2019, including 8 studies with a total of 267 patients, despite the promising initial results, show that there is no sufficient quality evidence to recommend routine implementation of preoperative bowel stimulation in clinical practice.

Based on studies such as the one published by Morgan et al. in 2020 [34], which demonstrated the important role of the intestinal microbiome in the development of microscopic colitis and the regression of inflammation after reconstructive surgery, we considered that stimulation with probiotics prior to closure of the protective ileostomy would allow the altered autoimmune function to be normalized, restore homeostasis, and reduce mucosal inflammation, achieving the reduction or disappearance of the macroscopic and microscopic changes associated with it. This initial hypothesis seems to correlate with the results we have obtained, achieving a remission in endoscopic and histological findings of DC in SG, as we can see in Figure 2 and Figure 3, where a significant remission in SG was observed compared to CG after stimulation with probiotics prior to closing the protective stoma. This remission in endoscopic and histological findings had already been studied in previous clinical trials, in experimental models and phase III studies, through stimulation with different substances such as nutritional solutions, fecal transplantation, short chain fatty acid enemas (SCFA) such as butyrate, 5-aminosalicylate acid (5-ASA), glucocorticoids, sucralfate and N-acetylcysteine among others, with different results [15,16,17,18,35,36,37,38,39,40]. Most of them showed inconsistent results, although there seems to be a decrease in intestinal mucosa inflammation, remission epithelial lesions and neutrophils.

**Figure 4:**
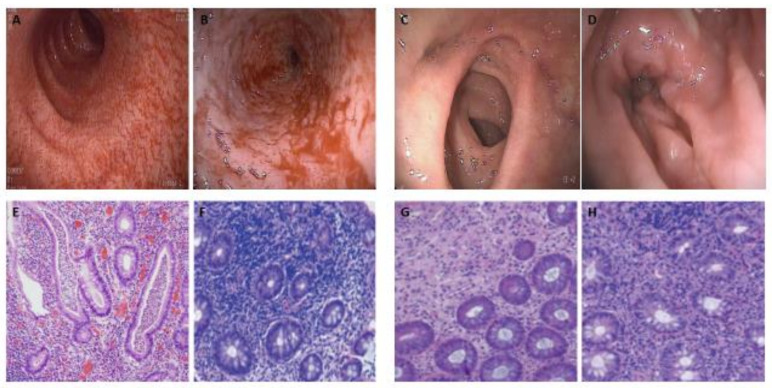
Endoscopic and histopathological findings of diversion colitis. (**A**–**B**): Pre-stimulation colonoscopy showed easily hemorrhagic mucosa, with edema, spontaneous bleeding, erythema and stenosis (**B**). (**C**–**D**): Biopsies after probiotics stimulation demonstrated only low-grade inflammation and edema, with diffuse erythema (**D**), without erosions and friability mucous. (**E**–**F**): Pre-stimulation histopathological evaluation of colon biopsies with hematoxylin and eosin staining showed inflammation with lymphoplasmocytic infiltration, follicular lymphoid hyperplasia, crypt abscesses, and crypt architecture distortion. (**G**–**H**): Biopsies after probiotics stimulation demonstrated a low-grade inflammation with reduced crypt abscesses, lymphoplasmocytic infiltration, follicular lymphoid hyperplasia, and crypt architecture distortion.

The results of this study, at the macroscopic level, demonstrated a significant decrease in all endoscopic findings in SG compared to CG, with a strong correlation between stimulation of the efferent loop with probiotics and a decrease in macroscopic findings on colitis. It was worth highlighting a notable decrease in the severity of the edema and mucous erythema, turning from severe or moderate to mild or simply disappearing, a fact that we relate to the anti-inflammatory effect of probiotics, and a complete disappearance of the ulcers in the SG after the stimulation phase, also with an intense correlation, as we can see in Figure 2 and Figure 4. Ulcers associated with DC in humans were an inconsistent finding. There seems to be an association with late structural changes on the superficial epithelium [15,16]. In our study, the patients with mucous ulcers were those living more time with the stoma. These findings were consistent with previous observations made in humans and animal models [37,38].

Morphological studies about colonic biopsies in patients with ileostomy have shown chronic inflammation in more than 50% of the samples, with an increase in lymphoid tissue in 100% of the cases [15,40]. The most significant histologic finding in DC is lymphoid follicular hyperplasia, which was present in experimental models and patients 60 days after the creation of the stoma [20], with infiltration of the mucosa by lymphocytes that can be from mild to severe. Pacheco et al. described lymphoid follicular hyperplasia in 100% of the cases in animal models, and crypt atrophy in approximately 24% after six weeks of the stoma creation and 42% after 17 weeks [37,38]. In our study, histopathological changes were characterized, as in previous studies, mainly by lymphoid follicular hyperplasia and lymphocytic infiltration of the lamina propria, with destructuring of the crypt architecture and cryptitis in those patients with a longer time since the stoma was created. A significant remission in microscopic alterations in SG compared to CG was seen, with an intense correlation between stimulation of the efferent loop with probiotics and the decrease or disappearance of histological alterations, as we can see in Figure 3 and Figure 4. As we have commented previously, these findings could be explained by the ability of probiotics to interact with the intestinal mucosa, decreasing the molecular production of pro-inflammatory substances, and thereby reducing the migration capacity of inflammatory cells to the lamina propria, such as lymphocytes, eosinophils, and plasma cells.

The observations presented here demonstrate that the decrease in endoscopic and histological findings associated with the decrease in inflammation means that stimulation of the efferent loop of the ileostomy with probiotics can be an alternative treatment in patients with symptomatic DC who are not candidates for reconstructive surgery as a treatment to resolve the colonic inflammation. Finally, the stimulation of the efferent loop with probiotics can be an alternative treatment to resolve the inflammation in patients whose surgical option is not feasible or available.

There are few studies on endoscopic and histopathological DC after reconstructive surgery, with limited information on follow-up. A Korean study published by Son et al. [27] found an incidence of symptomatic DC in 63.3% of patients with ileostomy, remaining in 46.6% six months after its closure. Szczepkowski et al. [15] observed in their 2017 study a persistence of macroscopic and microscopic inflammation in patients with a diagnosis of DC at three months and five years after the reconstructive surgery. In this study, limited by a small sample size, the endoscopic and histological manifestations of DC decreased after reconstructive surgery, observing reappearance of the inflammation in the colonic mucosa in the long term. In the short-term follow-up after reconstructive surgery, a significant correlation was observed between stimulation of the efferent loop with probiotics and the disappearance of the symptoms associated with DC in SG compared to CG, observing a persistence of DC in the CG of around 30% after closure of the ileostomy.

## 5. Conclusions

Therefore, we can conclude that stimulation of the efferent loop with probiotics seems to have a reducing effect on the endoscopic and histopathological alterations in DC and consequently, this procedure can be an alternative treatment for patients where surgical option is not feasible or available. Nevertheless, we will need more multicenter studies based on macroscopic and microscopic changes after stimulation prior to closure ileostomy to support these conclusions.

## Figures and Tables

**Table 1:** Demographics and clinics characteristics in Stimulated and Control group.

	Stimulated Group (*n* = 34)	Non Stimulated Group (*n* = 35)	*p*
Demographics			
Age (years)	65 (45–81)	68 (41–80)	0.421
Sex ratio (M:F)	23:11	25:10	0.170
BMI (kg/m^2^)	23.5 (21.6–32.6)	27.6 (18.8–40.2)	0.091
ASA			0.483
ASA I-II	31	30	
ASA III	3	5	
Smoker/nonsmoker	20/14	23/12	0.826
Time between surgeries (months) *	12 (8–37)	9 (6–32)	0.813
Clinic:			
Asymptomatic	10 (29.4%)	14 (40%)	0.309
Abdominal pain	15 (44.1%)	18 (51.4%)	0.402
Tenesmus	5 (14.7%)	2 (5.7%)	0.702
Mucous Discharge	21 (61.7)	14 (40%)	0.117
Rectorrhagia	2 (5.9%)	4 (11.4%)	0.668

BMI: body mass index; ASA: American Society of Anesthesiologists Classification; * Time from creation of stoma to the closure of the protective ileostomy.

## Data Availability

All data generated in this study is shown in this article, its tables, and figures.
